# Editorial: Best Practice Approaches for Mixed Methods Research in Psychological Science

**DOI:** 10.3389/fpsyg.2020.590131

**Published:** 2020-12-09

**Authors:** M. Teresa Anguera, Angel Blanco-Villaseñor, Gudberg K. Jonsson, José Luis Losada, Mariona Portell

**Affiliations:** ^1^Faculty of Psychology, Institute of Neurosciences, University of Barcelona, Barcelona, Spain; ^2^Faculty of Psychology, University of Barcelona, Barcelona, Spain; ^3^Human Behavior Laboratory, School of Health Sciences, University of Iceland, Reykjavík, Iceland; ^4^Department of Psychobiology and Methodology of Health Sciences, Universitat Autònoma de Barcelona, Cerdanyola del Vallès, Spain

**Keywords:** QUAL-QUAN integration, symmetry, quantitizing, qualitizing, record transformation, mixed methods

## Introduction

Mixed methods research burst onto the scene around the beginning of the second millennium. After decades of intense dispute between those who preferred the qualitative perspective and their quantitative counterparts—with both sides having grown deeply entrenched in their respective views—a complementary approach promising the possibility of integration had finally been proposed. By that time, however, the vast majority of researchers had committed to one stance or the other; very few of us argued that the two approaches could be complementary.

Since then, the number of publications, scientific meetings and other activities devoted to the mixed methods approach has increased exponentially throughout the world. For us, there are two definitions specially relevant. Teddie and Tashakkori (2010) defined mixed methods research as “research design using qualitative and quantitative data collection and analysis techniques in either parallel or sequential phases” (p. 11). And Johnson et al. (2007) say that “Mixed methods research is the type of research in which a researcher or team of researchers combines elements of qualitative and quantitative research approaches (e.g., use of qualitative and quantitative viewpoints, data collection, analysis, inference techniques) for the broad purposes of breadth and depth of understanding and corroboration” (p. 123). Moreover, Johnson et al. (2007) have listed and analyzed 19 definitions of mixed methods, and the authors that have worked on this topic as a part of a big community. The expansion of mixed methods in the scientific community has been expanding rapidly.

At a substantive level, we are pleased to see that a growing number of fields are generating mixed methods research, and we are eager to assist in promoting this trend. However, the field has experienced some “growing pains”: a certain degree of heterogeneity in terms of approaches, differences of opinion regarding certain conceptualizations (for example, mixed methods vs. multi-methods), numerous design taxonomies, multiple ways of integrating qualitative and quantitative elements, and various positions on how best to overcome the enduring lack of symmetry between qualitative and quantitative aspects. The methodological and substantive spectrum is vast and broad, possibly because the mixed methods approach has become “obligatory” for much research, not only in psychology but in practically all branches of the social sciences.

Our proposal for delineating between mixed methods and multimethods has been presented in a previous work (Anguera et al., 2018). We believe that a study will be multimethod when, driven by a common overall research goal, it uses a series of complementary methodologies, chosen according to a given criterion. According to our proposal, whether it has a predominantly qualitative or quantitative nature has no bearing on its consideration as a multimethod study. By contrast, the essence of mixed methods studies is that they contain qualitative and quantitative components that must be integrated to ensure the mixing of the information they carry. Combining and integrating quantitative and qualitative data in the same study, however, poses numerous challenges, and attempts have been made in recent years to untangle this *Gordian knot*, generating and developing strategies for successfully integrating qualitative and quantitative data.

The aim of this Research Topic is to present a selection of studies whose methodological approaches include, as a central element, aspects related to the *Gordian knot* of mixed methods, that also incorporate secondary—but no less important—elements such as dataset transformation, analytical techniques and data integration, as well as studies in which systematic observation is used as a mixed method in itself. The Research Topic has promoted a transparent presentation of the mixed approach used to develop the conceptual, methodological or application-related contribution of each article. This transparency will enable other researchers to critically appraise and replicate the methods used.

The 32 articles that make up the Research Topic *Best Practice Approaches for Mixed Methods Research in Psychological Science*, with contribution from 121 authors, are organized from a substantive point of view in different criteria, although each of the published articles could have been “classified” from several points of view.

## Methodological Developments

It is important to highlight the contributions made in the articles published in this Research Topic from the methodological criteria, given the conceptual amplitude of the *mixed methods* topic and its repercussions in applied studies.

We distinguish different procedural orientations, which could be structured around different facets, such as conceptual, technological, methodological, psychometric, and teaching.

Schoonenboom's work focuses centrally on case development from the perspective of *mixed methods*, conceptually showing how to save the successive controversies that may arise, to later develop subcases, and finally, a moderate case.

If we look from a technological perspective in the *mixed methods*, we locate the article by Müller et al., which focuses on the study of sensors to study social processes, which provide quantitatively and qualitatively treatable data. From a laboratory setting, Casarrubea et al. deepens in reflections about the meaning of “the qualitative” and “the quantitative.” Zurutuza et al., in a telemetric study, use data from GPS technology, now expanding.

If we refer to computer programs used, we highlight the LINCE recording program (see Alcover et al.; Aranda et al.; Casal et al.; Escolano-Pérez, Acero-Ferrero et al.; Escolano-Pérez, Herrero-Nivela et al.; Maneiro et al.; Portell et al.; Prat et al.; Terrenghi et al.), the SAGT generalizability analysis program (see Vázquez-Diz et al.; Vázquez-Diz et al.), the GSEQ record and analysis program (see Del Giacco et al.; Escolano-Pérez, Acero-Ferrero et al.; Escolano-Pérez, Herrero-Nivela et al.; Morales-Sánchez et al.; Portell et al.; Venturella et al.), the HOISAN record and analysis program (see Alcover et al.; Camerino et al.; Del Giacco et al.; Escolano-Pérez, Herrero-Nivela et al.; Menescardi et al.; Morales-Sánchez et al.; Portell et al.; Vázquez-Diz et al.; Vázquez-Diz et al.), and the THEME analysis program (see Brill and Schwab; Camerino et al.; Casarrubea et al.; Escolano-Pérez, Herrero-Nivela et al.; Hunyadi; Morales-Sánchez et al.; Portell et al.; Prat et al.; Szekrényes). Furthermore, we highlight the studies by Suárez et al. and Terrenghi et al., in which the DRAGON program has been used for the transcription of interviews; that of Morales-Sánchez et al., where the FACE READER program has been used to obtain data on facial expressions, ALCESTE in the study by Rodríguez-Naveiras et al. for text analysis; ATLAS.ti in Suárez et al. also for text analysis; AMOS in the article by Teques et al. for the analysis of structural equations; MATLAB in Menescardi et al., and the WEKA tool to materialize data mining in the article by Pastrana et al.. In turn, the SPSS has been used in Aranda et al., Maneiro et al., Rodríguez-Naveiras et al., and R in Casal et al..

From a methodological approach, Magnusson's seminal work shows how T-Pattern Analysis (TPA) passes repeatedly between qualitative and quantitative analysis, and precisely this analysis technique has allowed the performance of multiple *mixed methods* studies, be treated in a unique way, or combined with others, with the analysis of polar coordinates, as in the work of Portell et al..

There are several articles published in this Research Topic that have used the TPA, and with a methodological purpose rather than application. Hunyadi's article is an exponent of the great possibilities in the field of communication understood in a multimodal way, through the HuComTech project, and that of Szekrényes, which technologically allows starting records in ELAN to analyze the data with THEME.

The essential *desideratum* of *mixed methods* lies in the use of data of diverse nature, and the study by Brill and Schwab uses data from self-reports (from questionnaires) and videographic recording of behavior, in addition to content analysis. Likewise, Teques et al. start from the data of self-reports and observational records. For their part, in Prat et al. data were obtained from observational instruments and Likert scales. In Suárez et al. interviews, questionnaires, and observational records are used. And in Terrenghi et al. videographic recordings, self-reports, manual registration, questionnaires, and focus groups were the chosen data gathering methods.

In recent years, consideration of the observational methodology began as mixed method itself (Anguera and Hernández-Mendo, 2016; Anguera et al., 2017), and has expanded rapidly, as this Research Topic attests. Among the 32 published articles, there are 17 that use observational methodology, and from this point of view, the great macro-stages that characterize the process are expressed through the QUAL-QUAN-QUAL, which allows qualitative data to be transformed into other types, also qualitative, but in such a way that they can be treated quantitatively, and then interpreted qualitatively (Anguera et al., 2020; Anguera et al., in press). This interpretation of *mixed methods* is strongly supported by the words of Creswell and Plano Clark (2007), when referring to *connecting* as a way of integration between qualitative and quantitative elements. The articles consisting of empirical studies found in this block are the following, in alphabetical order of the first author: Alcover et al., Aranda et al., Camerino et al., Casal et al., Del Giacco et al., Escolano-Pérez, Acero-Ferrero et al., Escolano-Pérez, Herrero-Nivela et al., Maneiro et al., Menescardi et al., Morales-Sánchez et al., Portell et al., Prat et al., Suárez et al., Vázquez-Diz et al., Vázquez-Diz et al., Venturella et al., and Zurutuza et al..

Reflecting on the transit that has been carried out in certain areas, from controlled clinical trials, considered as mono-method, to *mixed methods*, there is a conceptual path that is emphasized by Carey et al., and paying special attention to causation and operationalization.

With a clearly psychometric interest, the works of Timoszyk-Tomczak et al., and Llistosella et al. were published, about the adaptation of a measurement instrument.

From the point of view of data analysis, the following techniques have been used in the empirical studies of this Research Topic: TPA (9), analysis of polar coordinates (8), analysis of generalizability (4), lag sequential analysis (3), analysis of variance (3), and, to a lesser extent, comparison of proportions, Student's t, Pearson's correlation analysis, factor analysis, principal component analysis, cluster analysis, logistic regression, structural equation models, and decision tree.

And we end this block with teaching on *mixed methods*, with the work of Roberts et al., which advocates that the teaching of *mixed methods* be carried out by insisting from the beginning on the integration of qualitative and quantitative methods, instead of doing it separately and sequentially.

## Areas of Application

The studies that we publish in this section stand out for both substantive and procedural aspects within *mixed methods*, but we have considered that the emphasis that they represent at the level of application areas was the most important.

In decreasing order, there are 11 articles in the field of sport, 7 in school-education, 4 in clinical psychology, 2 in conversation analysis, and one in each of the following fields: occupational health, the media, feeding behavior in rats, resilience, organizational psychology, time, and teaching. Furthermore, there is one that is purely methodological, and does not refer to any substantive scope.

## Conclusions

In short, the articles included in the Research Topic make up a broad spectrum.

As Editors of this Research Topic, we want to express the satisfaction that comes from having the opportunity to offer the materialization of new studies in the exciting field of mixed methods to the scientific community.

The Research Topic proposal has been motivating, exciting and satisfying, as well as the highest level of acceptance of the originals. Regarding the management, the originals of the 32 articles that make up this Research Topic were published between January 2019 and July 2020. 48 manuscripts were sent; therefore, the acceptance percentage was 66.6%.

## Author Contributions

All authors listed have made a substantial, direct and intellectual contribution to the work, and approved it for publication.

## Conflict of Interest

The authors declare that the research was conducted in the absence of any commercial or financial relationships that could be construed as a potential conflict of interest.
